# A Multidimensional Connectomics- and Radiomics-Based Advanced Machine-Learning Framework to Distinguish Radiation Necrosis from True Progression in Brain Metastases

**DOI:** 10.3390/cancers15164113

**Published:** 2023-08-15

**Authors:** Yilin Cao, Vishwa S. Parekh, Emerson Lee, Xuguang Chen, Kristin J. Redmond, Jay J. Pillai, Luke Peng, Michael A. Jacobs, Lawrence R. Kleinberg

**Affiliations:** 1 Department of Radiation Oncology and Molecular Radiation Sciences, Johns Hopkins University School of Medicine, Baltimore, MD 21231, USA; 2 Department of Radiation Oncology, Dana-Farber/Brigham and Women’s Cancer Center, Harvard Medical School, Boston, MA 02115, USA; 3 Russell H. Morgan Department of Radiology and Radiological Science, Johns Hopkins University School of Medicine, Baltimore, MD 21231, USA; 4 University of Maryland Medical Intelligent Imaging (UM2ii) Center, Department of Diagnostic Radiology and Nuclear Medicine, University of Maryland School of Medicine, Baltimore, MD 20201, USA; 5 Department of Radiation Oncology, University of North Carolina, Chapel Hill, NC 27514, USA; 6 Division of Neuroradiology, Mayo Clinic, Rochester, MN 55905, USA; 7 Department of Neurosurgery, Johns Hopkins University School of Medicine, Baltimore, MD 21231, USA; 8 Department of Diagnostics and Interventional Imaging, McGovern Medical School, Houston, TX 77030, USA

**Keywords:** radiomics, connectomics, radionecrosis, radiosurgery, brain metastases, machine learning

## Abstract

**Simple Summary:**

Diagnosing true progression (TP) versus radiation necrosis (RN) in brain metastases treated with stereotactic radiosurgery is a significant clinical challenge that can lead to delays of care or unnecessary neurosurgical procedures. We implemented a novel machine-learning framework that uses both multiparametic radiomics (mpRad) and tumor connectomics analysis to probe the textural properties and structural networks within radiographically progressive lesions, respectively. Our predictive model was able to distinguish histopathologically proven cases of TP from RN with excellent discrimination and may ultimately serve as a useful tool to inform clinical decision making.

**Abstract:**

We introduce tumor connectomics, a novel MRI-based complex graph theory framework that describes the intricate network of relationships within the tumor and surrounding tissue, and combine this with multiparametric radiomics (mpRad) in a machine-learning approach to distinguish radiation necrosis (RN) from true progression (TP). Pathologically confirmed cases of RN vs. TP in brain metastases treated with SRS were included from a single institution. The region of interest was manually segmented as the single largest diameter of the T1 post-contrast (T1C) lesion plus the corresponding area of T2 FLAIR hyperintensity. There were 40 mpRad features and 6 connectomics features extracted, as well as 5 clinical and treatment factors. We developed an Integrated Radiomics Informatics System (IRIS) based on an Isomap support vector machine (IsoSVM) model to distinguish TP from RN using leave-one-out cross-validation. Class imbalance was resolved with differential misclassification weighting during model training using the IRIS. In total, 135 lesions in 110 patients were analyzed, including 43 cases (31.9%) of pathologically proven RN and 92 cases (68.1%) of TP. The top-performing connectomics features were three centrality measures of degree, betweenness, and eigenvector centralities. Combining these with the 10 top-performing mpRad features, an optimized IsoSVM model was able to produce a sensitivity of 0.87, specificity of 0.84, AUC-ROC of 0.89 (95% CI: 0.82–0.94), and AUC-PR of 0.94 (95% CI: 0.87–0.97).

## 1. Introduction

It has been estimated that 20–40% of cancer patients will develop brain metastases during their disease course [1]. Stereotactic radiosurgery (SRS) is a technique used to deliver highly conformal radiation doses to either intact brain metastases or the postoperative bed, with reported local control rates ranging from 70–95% depending on the lesion size [2]. This approach, in contrast to whole-brain radiation therapy, is typically associated with minimal impact on cognitive function. As such, the utility of SRS has increased in modern cancer care, especially as improved systemic therapy options have increased the duration of survival for patients with metastatic disease. Despite the efficacy of SRS in controlling intracranial metastases, a subset of treated lesions will recur, noted either on surveillance MRI or upon workup of evolving neurologic symptoms.

A significant diagnostic challenge faced in this setting is the concept of radiation necrosis (RN), a local inflammatory treatment effect that is indistinguishable from tumor progression on conventional, standard-of-care MRI. The incidence of RN is estimated to be 5–25% after SRS [3,4,5], with diagnosis typically relying on close follow-up imaging obtained over a period of months. Approximately 40% of patients with progressive post-treatment imaging changes will have necrosis, whereas the remainder develop progressive tumors, leading to uncertainty about the optimal management of changes in conventional imaging.

There have been varied efforts to use machine learning and advanced image analysis techniques to tackle this diagnostic issue, most commonly with MRI-based radiomics [6,7,8,9,10,11]. Radiomics is defined as the textural analysis of images to infer tissue pathology [12]. However, radiomics typically summarizes textural features of individual voxels or simple relationships among neighboring voxels, which may fail to capture underlying structural properties within the region of interest. We introduce tumor connectomics [13], a novel MRI-based complex graph theory framework that describes the properties of the intricate network of voxels with similar characteristics, both within the tumor and the surrounding tissue, to distinguish pathologically proven TP vs. RN in radiographically progressive brain metastases that were treated with SRS.

## 2. Materials and Methods

**Figure 1:**
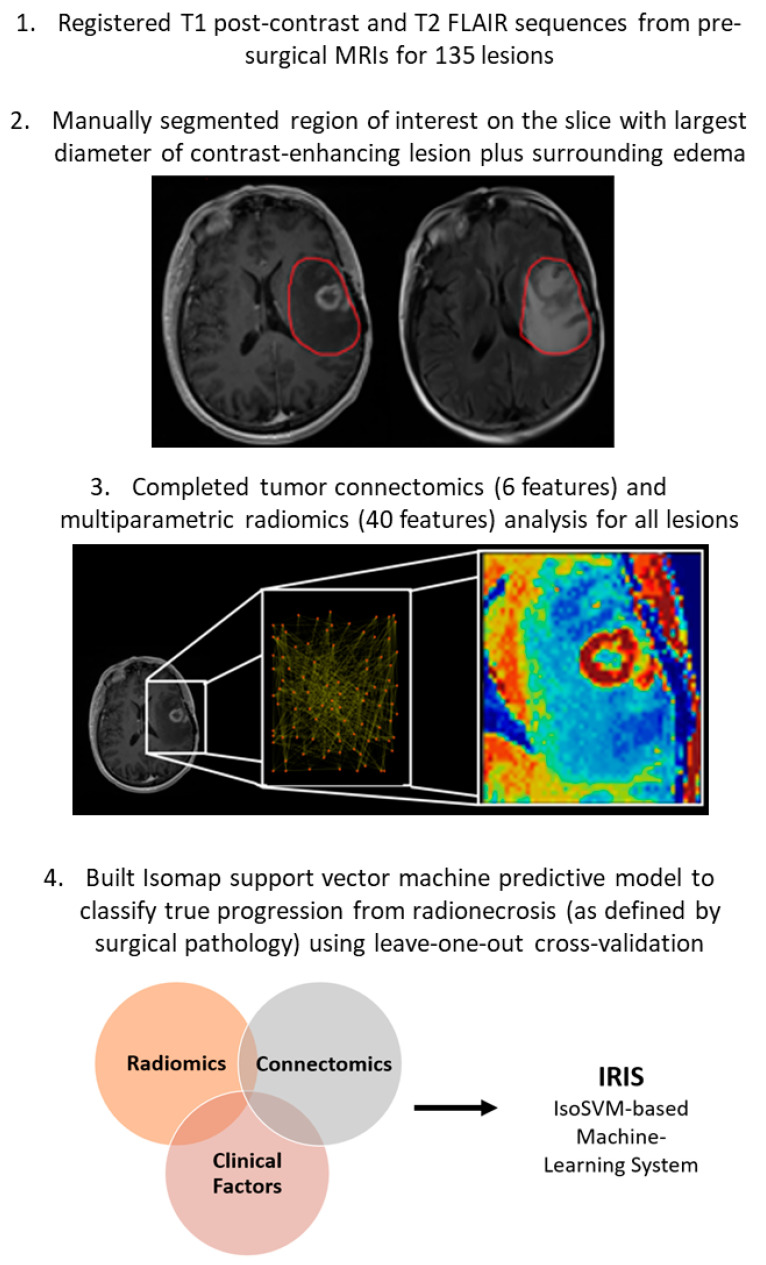
Graphical summary of methods.

The general methods of this study are summarized in graphical form in Figure 1.

### 2.1. Patient Inclusion and Clinical Factors

After Institutional Review Board approval, we retrospectively identified patients with primary solid tumors who had a history of single- or multi-fraction SRS between 2010 and 2020 and ultimately underwent pathological assessment (i.e., neurosurgical resection or biopsy) to histopathologically evaluate radiographic progression. The presence of any foci of active tumor cells within the final pathology report was classified as TP. All lesions included in this series were treated with SRS, and lesions that received another radiation course in addition to SRS were not excluded (i.e., repeat SRS, whole-brain radiation therapy (WBRT), or prophylactic cranial irradiation (PCI)).

Eligible patients were retrospectively chart-reviewed using a combination of the electronic medical record and radiation therapy treatment summary documents to record the following five clinical and treatment factors included in our model-building analysis: primary histology, history of multiple radiation courses to the area of suspected radiographic progression, biologically effective dose (BED), prescription isodose line, and planning target volume (in cc). The BED was calculated according to the following equation: BED = D × (1 + [d/(α/β)]), where D is the total dose in Gray (Gy), d is the dose per fraction, and α/β is assumed to be 10.

### 2.2. Imaging Feature Extraction

#### 2.2.1. Region of Interest

The MRI sequences used for the analysis were the T1-weighted post-contrast (T1C) and T2-weighted fluid attenuated inversion recovery (T2 FLAIR) sequences obtained prior to surgical assessment. Using the Velocity software (Varian Medical Systems), the T2 FLAIR images were automatically rigidly registered to the T1C sequences via alignment with the bony anatomy. The single slice on the T1C sequence with the largest diameter of the contrast-enhanced lesion was identified, and the region of interest (ROI) was contoured to encompass the T2 FLAIR hyperintensity associated with that slice.

#### 2.2.2. Tumor Connectomics

Tumor connectomics is a theoretical graph technique developed to model the complex tumoral and surrounding tissue network and investigate the biological organizational structure within the tumor network [13]. We implemented the previously described tumor connectomics framework (TCF) to model the brain metastasis network (BMN). The BMN was modeled using the following steps:

1. In the first step, the MRI image intensities across both the T1C and corresponding T2 FLAIR images were normalized to values between 0 and 1 (double-precision) to avoid any bias from intensity values.
2. The second step involved computing an inter-voxel pair-wise Euclidean distance matrix. A threshold of 0.1 was empirically selected to transform the Euclidean distance into geodesic distance matrix using Dijkstra’s algorithm [14].
3. The geodesic distance matrix was then evaluated to extract six different quantitative graph metrics of degree centrality, betweenness centrality, eigenvector centrality, node strength, average path length, and clustering coefficient.

#### 2.2.3. Multiparametric Radiomics

Multiparametric radiomics (mpRad) is a radiomics technique capable of quantifying texture from a multisequence or multiparametric imaging dataset. This is in contrast to conventional radiomics techniques that are only capable of quantifying texture in single images or volumes using a logarithmic transformation. The mpRad technique uses a tissue signature of the different tissue types instead of voxel intensities for quantifying texture. We extracted mpRad features from the T1C and T2 FLAIR sequences using the following steps:

1. In the first step, the MRI image intensities across both sequence images were normalized to values between 0 and 1 (double-precision) to avoid any bias from the intensity values.
2. The second step involved the extraction of tissue signature first-order statistics (TSFOS), tissue signature probability matrix (TSPM), and tissue signature co-occurrence matrix (TSCM) features, resulting in a total of 40 unique mpRad features. These features consisted of 2 TSPM, 15 TSFOS, and 23 TSCM features, as previously described [15].

### 2.3. IsoSVM Classification

Our feature extraction method extracted a total of fifty-one different features. These included features modeled from three different domains: clinical/treatment features (5 features), tumor connectomics features (6 features), and multiparametric radiomics features (40 features). All analyzed features are listed in Supplementary Table S1. The predictive model for distinguishing TP from RN was developed using the Isomap support vector machine (IsoSVM) algorithm within our in-house platform called the IRIS (Integrated Radiomics Informatics System) [16,17,18]. The IsoSVM algorithm is a nonlinear classifier that first uses the Isomap algorithm to map all the features from a nonlinear to a linearly separable space and then trains a linear SVM in the transformed space.

Because SVM is a linear binary classification algorithm that attempts to create a linear hyperplane that best separates the different groups, the application of the Isomap algorithm prior to SVM transforms the data in a multidimensional nonlinear feature space to a linearly separable space [16,17]. Mathematically, the trained IsoSVM classifier uses the following equation to make a prediction for a test patient with the multidimensional feature space (connectomics, radiomics, and clinical features) represented by *x*:

(1) $$f\left(x\right)=\sum_{i=1}^{N}\alpha_{i}y_{i}<\phi \left(x_{i}\right),\phi \left(x\right)>+b$$

where φ() is the Isomap transformation function that maps the multidimensional feature space into a linearly separable space, N is the number of patients in the training set, $\alpha_{i}$ are the Lagrange multipliers, $x_{i}$ represents the feature signatures of training set patients, and x represents the feature signature of the test patient.

The IsoSVM algorithm was trained and evaluated using leave-one-out cross-validation. The SVM training hyperparameters were optimized using a grid search, and the optimal feature set was obtained using forward selection with the area under the receiving operating characteristic curve (AUC-ROC) and the Matthews correlation coefficient (MCC) as the optimizing metrics.

Machine-learning classifiers tend to overfit and classify the over-represented class in order to optimize the accuracy or the performance. Therefore, we used two important features in our training to avoid this overfitting.

1. We used a combination of the AUC-ROC and MCC as our optimization metric instead of just the conventionally used AUC-ROC. This is because the MCC metric is a balanced statistical metric that produces a good score only when the prediction accuracy across all classes is high, ensuring that the classifier does not overfit towards the over-represented class [13,19].
2. We used differential misclassification weighting during model training to identify the optimal misclassification ratio that produces a balanced performance [16,17]. We varied our misclassification ratio of RN:TP from 1:1 to 5:1 during our grid search to identify the optimal misclassification ratio.

### 2.4. Statistical Analysis

Summary statistics (mean and standard deviation of the mean) were computed for each of the graphic metrics analyzed. Variables were compared between the RN and TP groups using the two-tailed *t*-test for continuous variables and the Chi-squared test for categorical variables. We computed the area under the receiver operating characteristic (ROC) curve (AUC-ROC) and the area under the precision recall (PR) curve (AUC-PR) to assess model performance.

## 3. Results

In total, 135 lesions in 110 patients were analyzed, including 43 cases (31.9%) of pathologically proven RN and 92 cases (68.1%) of TP. The median elapsed time between the date of MRI used for imaging feature extraction and the date of surgical sampling was one day (IQR: 1-1), with only 9 of 135 (6.7%) cases where imaging preceded pathological assessment by more than five days. Table 1 summarizes the key clinical characteristics of the total cohort, as well as comparative summary statistics for the RN and TP groups. The distribution of tumor histology, proportion of patients receiving another course of radiation therapy in addition to SRS, mean planning target volume (i.e., proxy for lesion size), and mean prescription isodose line (i.e., proxy for heterogeneity of radiation dose within the target) were not statistically different between the RN and TP groups. However, the mean biologically effective dose (which helps normalize radiation dose across different radiation treatment schedules) was statistically higher in the RN group, although an absolute value difference in the mean BED10 was just 3.5 Gy (48.3 vs. 44.8 Gy BED10; p = 0.03). Lastly, the mean time from SRS to definitive pathologic diagnosis was significantly longer in the RN cohort compared to the TP cohort (15.2 vs. 10.8 mo; p = 0.02).

The most common primary histologies were NSCLC, breast, melanoma, and SCLC, altogether comprising 83% of the lesions analyzed. One-fifth of the lesions received a separate course of RT in addition to SRS, the majority (75.8%) of which was WBRT or PCI. Approximately half (54.4%) of the patients received single-fraction SRS, with prescribed dose ranging from 14–20 Gy. Of the RN cases, 32.6% underwent surgery within 9 months of SRS. Meanwhile, 52.2% of TP cases underwent surgery within 9 months following SRS.

The three top-performing connectomics features and ten mpRad features comprised the final IsoSVM model to distinguish RN from TP. These imaging features included: (1) multidimensional entropy, (2) multidimensional uniformity, (3) variance, (4) energy, (5) cluster tendency, (6) cluster prominence, (7) degree centrality, (8) standard deviation, (9) cluster shade, (10) betweenness centrality, (11) contrast, (12) eigenvector centrality, and (13) dissimilarity. The three centrality measures were the imaging features derived from the TCF. Meanwhile, none of the clinical features investigated were determined in the model-building process to be a top-performing variable warranting inclusion in the final IsoSVM predictive model.

**Figure 2:**
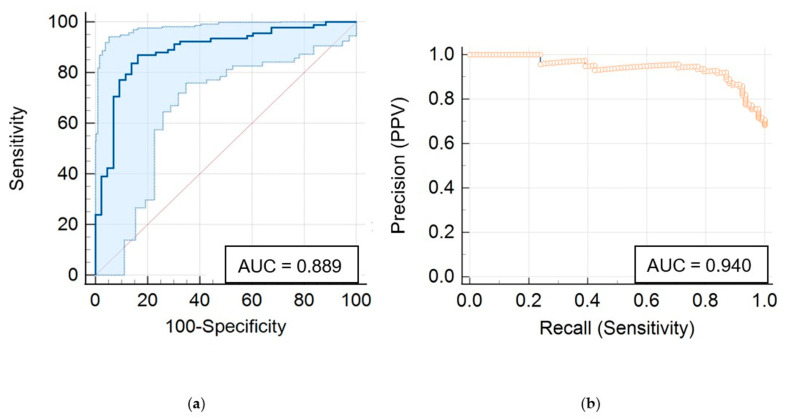
Performance of IsoSVM classifier model for distinguishing pathologically proven radiation necrosis vs. true progression with (**a**) receiver operator characteristic analysis and (**b**) precision recall analysis.

Our combined mpRad and TCF predictive model was found to have a sensitivity of 87%, specificity of 84%, AUC-ROC of 0.89 (95% CI: 0.82–0.94), and AUC-PR of 0.94 (95% CI: 0.87–0.97) for distinguishing pathologically proven RN vs. TP (Figure 2).

## 4. Discussion

As the metastatic state in stage IV cancer patients continues to be extended by advances in systemic therapies, the role of radiosurgery will likely continue to grow in parallel. Therefore, addressing the significant diagnostic challenge of distinguishing RN vs. TP after SRS for brain metastases will be increasingly important, as the gold standard of surgical sampling can be unnecessarily invasive in cases of RN or lead to delays in care if cases of TP are serially monitored with imaging (i.e., delays to repeat radiation therapy, surgical resection, or changes in systemic therapy). We present a novel multidimensional, multidomain machine-learning-based imaging analysis model that has the potential to distinguish RN from TP with excellent discrimination. In the largest cohort to date of pathologically proven RN vs. TP, we report an AUC-ROC of 0.89 and AUC-PR of 0.94.

We build upon our previous work in radiomics [15,20] to add the concept of tumor connectomics to the brain metastasis literature. While radiomics classically focuses on imaging features on a per-voxel basis, tumor connectomics characterizes the network of structural connections within the tumor and surrounding tissue. We previously reported the potential for tumor connectomics analysis to predict benign vs. malignant lesions in gliomas, breast, and prostate [4]. We hypothesized that this approach could be useful in distinguishing active tumors, regardless of histology, from radiation necrosis in the setting of irradiated brain metastases.

With the addition of the novel tumor connectomics framework to mpRad, we are able to achieve a sensitivity of 87% and specificity of 84% in distinguishing RN from TP using just two standard-of-care sequences obtained from diagnostic MRI. This is opposed to other studies that rely on advanced, nonstandard imaging such as PET or SPECT, with varying degrees of success [9], [21,22,23,24,25]. To put our model’s performance in context, a recent meta-analysis summarizing seven studies that applied MRI radiomics techniques to classifying TP after SRS for brain metastases found a pooled sensitivity and specificity of 77% and 74% [26]. However, a weakness of the included studies is that the determination of necrosis was largely based upon clinician or investigator assessment of standard imaging follow-up and not pathologic confirmation.

With respect to clinical factors, our univariable analysis (Table 1) suggests that lesions ultimately found to be TP, on average, have a significantly shorter time interval from SRS to surgical sampling as compared with RN lesions. This is consistent with findings from prior reports [27,28]. One series showed that 52.5% of lesions histopathologically assessed prior to 9 months following SRS were demonstrated to be biopsy-proven TP, while only 6.3% of lesions were demonstrated to be TP after 9 months (*p* < 0.004) [28]. While our data suggest a similar trend, our findings are not nearly as dramatic using the same time point: 72.6% and 64.4% of lesions were found to be TP when surgically sampled before and after 9 months following SRS, respectively (*p* = 0.31). Indeed, this may not be a particularly reliable metric or a clinically relevant cutoff, as different institutions may have varying patterns and thresholds for surgical sampling vs. continued monitoring with serial imaging. As such, these data are difficult to interpret in the absence of information regarding symptomatic vs. asymptomatic radiographic progression. Interestingly, none of the clinical factors we investigated were high-performing enough to be selected by the machine-learning algorithm for the final model when compared with mpRad and TCF features. This is despite the long-established literature suggesting correlations between clinical factors (i.e., prior radiation exposure, histology, and planning target volume) and the risk of radiation necrosis [29,30,31]. Altogether, this suggests a potentially elevated role for the more objective advanced imaging analysis.

It is important to note that in this analysis, we segmented the region of interest to specifically include the areas of surrounding perilesional edema as represented by T2 FLAIR hyperintensity. Leeman et al. previously reported that a larger edema-to-lesion volume ratio was found to be predictive of RN as opposed to TP, with a particularly high positive predictive value of 92% when using a cutoff value of 10 for the edema-to-lesion volume ratio [27]. However, the sensitivity of this approach was not reported. Meanwhile, Dequesada et al. similarly demonstrated that the lesion quotient (ratio of the nodule on T2 imaging to the total enhanced area on T1 imaging) might be a promising diagnostic tool, with values < 0.3 suggesting RN and values > 0.6 suggesting TP [32]. However, identifying cutoff values again may have proven to be problematic, as these results could not be replicated in a follow-up study [32,33]. Nonetheless, these findings, in keeping with another study [34], point to the potential for an underlying clinical significance of the perilesional edema in distinguishing RN from TP. Our novel tumor connectomics framework and mpRad analysis may provide some additional insights. Interestingly, within the tumor connectomics features, mean scores for degree centrality, eigenvector centrality, and node strength were significantly higher in the RN cohort, while the average path length was lower. Meanwhile, TP cases demonstrated higher mean entropy and range scores within the mpRad metrics (Supplementary Table S1). This suggests that there is a greater degree of “connectivity” or similarity between the T1-enhanced and T2 FLAIR edema regions for RN cases than for TP cases. This may point to the fact that there is no discrete underlying tumor in RN, where imaging changes instead result from vascular changes and glial injuries that produce a more vasogenic edema [35]. With such an interpretation, the tumor connectomics framework may be reflecting the underlying biology and structural network of the region of interest as a continuous area of edema in the setting of a disrupted blood–brain barrier.

Our study has inherent limitations stemming from its retrospective nature. While our use of pathologic confirmation of RN vs. TP is a unique strength—given that diagnosis using serial imaging is often unreliable—we also recognize that the patients selected for surgical sampling may not be entirely representative of all patients who have suspected imaging progression. Moreover, pathologic confirmation is still susceptible to tissue sampling error when stereotactic needle biopsies are utilized (i.e., in cases where gross total resection is not technically feasible or favored), and this may confound our results by potentially underclassifying true progression cases. For our histopathological diagnosis, we also identified TP cases using strict criteria of any foci of an active tumor. This is likely an overly simplified classification model that may confound our model training, as (1) these foci may not be clinically significant or (2) these small active tumor deposits may not be captured on the single-slice ROI selected for feature extraction. As with all studies of this nature, this approach is at risk for model-overfitting to our institutional cases and would benefit from external validation. Moreover, we have yet to establish whether our final connectomics and mpRad predictive model could distinguish RN from TP prior to the point where surgical sampling is felt to be warranted, and a prospective study is needed for true validation to establish utility in guiding surgical decision making. Nonetheless, this study is a unique contribution to the literature in its introduction of the tumor connectomics framework, which is a valuable adjunct to the traditional radiomics approach. Future directions include the incorporation of advanced MRI sequences such as perfusion and diffusion imaging to further strengthen the predictive model.

## 5. Conclusions

In conclusion, a novel multidomain, multidimensional imaging tissue signature model built on routine MRI sequences was able to distinguish pathologically proven TP vs. RN following stereotactic radiation therapy to brain metastases with excellent discrimination. Validation using an independent data set is planned. This approach may help guide clinical decision making in selecting the patients most appropriate for surgical sampling vs. serial image monitoring in the common situation of radiographic progression after treatment.

## Figures and Tables

**Table 1:** Summary of lesion clinical characteristics.

	Entire Cohort (*n* = 135)	Radiation Necrosis Cohort (*n* = 43)	True Progression Cohort (*n* = 92)	*p*-Value
**Primary histology**				0.08
NSCLC	48 (35.6%)	10 (23.3%)	38 (41.3%)	
Breast	28 (20.7%)	10 (23.3%)	18 (19.6%)	
Melanoma	27 (20.0%)	14 (32.6%)	13 (14.1%)	
SCLC	9 (6.7%)	2 (4.7%)	7 (7.6%)	
Other	23 (17.0%)	7 (16.3%)	16 (17.4%)	
**RT in addition to SRS to the same area**	29 (21.5%)	11 (25.6%)	18 (19.6%)	0.43
**Mean SRS isodose line**, % (SD)	68.3 (8.8)	67.3 (9.7)	68.8 (8.4)	0.37
**Mean SRS BED10**, Gy (SD)	45.9 (8.2)	48.3 (8.9)	44.8 (7.7)	0.03
**Mean SRS PTV volume**, cc (SD)	11.0 (13.2)	9.8 (13.0)	11.5 (13.3)	0.50
**Mean time from SRS to surgery**, months (SD)	12.2 (10.0)	15.2 (10.2)	10.8 (9.7)	0.02

NSCLC = non-small-cell lung cancer; SCLC = small-cell lung cancer; RT = radiation therapy; SRS = stereotactic radiosurgery; SD = standard deviation; BED = biologically effective dose.

## Data Availability

Research data are not publicly available at this time. Anonymized data may be shared on reasonable request to the corresponding author and institutional agreement.

## Supplementary Materials

The following supporting information can be downloaded at: https://www.mdpi.com/article/10.3390/cancers15164113/s1, Table S1: Clinical, radiomics, and tumor connectomics parameters investigated in machine-learning model.
