# Supplementary material for: A Multidimensional Connectomics- and Radiomics-Based Advanced Machine-Learning Framework to Distinguish Radiation Necrosis from True Progression in Brain Metastases
Source: Cancers (Basel). 2023 Aug 15;15(16):4113. doi: 10.3390/cancers15164113 (PMC10452423; doi:10.3390/cancers15164113)
Supplement: Supplementary file 1 [file cancers-15-04113-s001.zip › cancers-2481398-supplementary.pdf]

Table S1: Clinical, radiomics, and tumor connectomics parameters investigated in machine-learning model

Feature	Radiation Necrosis		True Progression		t-test or Chi-squared test
	Mean	Std. Dev	Mean	Std. Dev	p-value
Clinical Factors					
Histology (NSCLC, breast, melanoma, SCLC, or other)	See Table 1				0.08
RT in addition to SRS to the same area	0.26		0.20		0.43
Biological equivalent dose	48.33	8.94	44.77	7.65	**0.03**
SRS isodose line	67.25	9.67	68.80	8.42	0.37
PTV (cc)	9.80	13.00	11.50	13.30	0.50
Connectomics Factors					
Degree Centrality	0.60	0.15	0.52	0.12	**0.00**
Node Strength	0.10	0.03	0.11	0.03	**0.03**
Betweenness Centrality	0.00042	0.00044	0.00031	0.00023	0.13
Average Path Length	1.55	0.31	1.67	0.27	**0.03**
Eigenvector Centrality	0.00073	0.00058	0.00047	0.00029	**0.01**
Radiomics Factors					
Energy	502.16	547.19	691.39	580.07	0.07
Entropy	5.15	0.30	5.15	0.29	0.98
Kurtosis	3.75	2.23	3.38	1.94	0.35
Maximum	0.67	0.15	0.74	0.15	**0.03**
Mean	0.30	0.06	0.31	0.07	0.30
Mean_absolute_deviation	0.09	0.03	0.10	0.03	0.16
Median	0.28	0.05	0.29	0.07	0.26
Minimum	0.06	0.04	0.04	0.03	0.05
Range	0.62	0.15	0.69	0.15	**0.01**
Root_mean_square	0.32	0.06	0.33	0.07	0.26
Skewness	0.72	0.57	0.61	0.52	0.31
Standard_deviation	0.11	0.04	0.12	0.04	0.16
Uniformity	0.04	0.01	0.04	0.01	0.67
Variance	0.01	0.01	0.01	0.01	0.28
Clonality	0.14	0.05	0.14	0.05	0.98
Autocorrelation1	443.85	185.41	484.29	222.46	0.27
Custer_Prominence1	176553.65	256947.01	205592.43	399959.03	0.61
Cluster_Shade1	2308.89	3468.81	2101.11	3700.78	0.75
Cluster_Tendency1	202.47	154.21	232.85	173.36	0.31
Contrast1	7.66	4.82	9.31	4.58	0.06
Correlation1	0.90	0.06	0.91	0.04	0.72
Difference_entropy1	1.68	0.24	1.76	0.23	0.06
Dissimilarity1	1.67	0.52	1.84	0.53	0.07
Energy1	0.01	0.01	0.01	0.01	0.15
Entropy1	5.10	0.46	5.31	0.49	**0.01**
Homogeneity11	0.56	0.07	0.54	0.07	0.15
Homogeneity21	0.51	0.08	0.49	0.09	0.15
Informational_measure_of_correlation_11	-0.34	0.07	-0.33	0.07	0.73
Informational_measure_of_correlation_21	0.93	0.04	0.93	0.03	0.39

Inverse_Difference_Moment_Normalized1	1.00	0.00	1.00	0.00	0.06
Inverse_Difference_Normalized1	0.98	0.01	0.97	0.01	0.07
Inverse_variance1	0.41	0.03	0.40	0.03	**0.02**
Maximum_probability1	0.04	0.02	0.04	0.03	0.41
Sum_average1	39.10	7.28	40.49	8.68	0.34
Sum_entropy1	3.72	0.28	3.84	0.26	**0.02**
Sum_variance1	1503.46	673.55	1646.97	810.24	0.28
Variance1	447.68	186.63	488.93	223.33	0.26
Clonality1	0.54	0.04	0.52	0.04	**0.01**
Multidimensional_entropy	7.46	0.57	7.80	0.64	**0.00**
Multidimensional_uniformity	0.01	0.00	0.01	0.01	0.41
